# Prevalence, Predictors and Point of View Toward Self-Medication Among Residents of Riyadh, Saudi Arabia: A Cross-Sectional Study

**DOI:** 10.3389/fpubh.2022.862301

**Published:** 2022-03-25

**Authors:** Basheerahmed Abdulaziz Mannasaheb, Sarah Abdulrahman Alajlan, Jaber Abdullah Alshahrani, Noordin Othman, Sultan Othman Alolayan, Mohammed Saleh Alamrah, Syed Mohammed Basheeruddin Asdaq, Awad Mohammed Al-Qahtani, Ibrahim Ahmed Shaikh, Mohammed Yahia Alasmary

**Affiliations:** ^1^Department of Pharmacy Practice, College of Pharmacy, AlMaarefa University, Riyadh, Saudi Arabia; ^2^Pharm.D Student, College of Pharmacy, AlMaarefa University, Riyadh, Saudi Arabia; ^3^Consultant Family Medicine, Armed Forces Hospitals, Khamis Mushayt, Saudi Arabia; ^4^Clinical and Hospital Pharmacy Department, College of Pharmacy, Taibah University, Al-Madinah Al-Munawwarah, Saudi Arabia; ^5^Department of Clinical Pharmacy, School of Pharmacy, Management and Science University, University Drive, Shah Alam, Malaysia; ^6^General Practitioner Physician at Almansk PHCC, Directorate of Health Affairs in Asir Region, Ministry of Health, Abha, Saudi Arabia; ^7^Department of Family and Community Medicine, College of Medicine, Najran University, Najran, Saudi Arabia; ^8^Department of Pharmacology, College of Pharmacy, Najran University, Najran, Saudi Arabia; ^9^Medical Department, College of Medicine, Najran University, Najran, Saudi Arabia

**Keywords:** self-medication, Riyadh (Saudi-Arabia), community, over the counter drugs, opinions

## Abstract

Irrational Self-Medication (SM) practice leads to incorrect diagnosis and is a risk factor for disease exacerbation and serious health consequences. Hence Responsible SM is vital for better health outcomes. In the present community-based study we explored the SM practice during the last 3 months, frequency, outcome, medications used, reasons influencing SM, source of the drug, and information. Data were analyzed using SPSS; chi-square test was performed to indicate significance, Odds ratio, Pearson correlation, univariant and multivariant regression analysis were performed to find out factors and predictors of SM. A total of 611 residents completed the survey. SM was practiced by 52.9% of participants during the last 3 months, with a frequency of one to two times. Headache (64.8%), pain (35.4%), fever/flu (31.4%), cold & cough (21.9%) and dysmenorrhea (20.9%) were illnesses managed using pain killers (75.9%), multivitamins (25.5%), anti-pyretic (24.7%) and herbal medicines (18.5%). Minor illness (67.9%), earlier experience (33.9%) and shortness of time to attend healthcare facilities (18.8%) were the reasons for practicing SM. Distance to healthcare and routine physical activity have significantly influenced the SM practice. Residents stated that inappropriate SM would lead to negative outcomes, including drug side effects (70%), Interaction (34.2%), poor treatment outcome (32.6%) and return of symptoms (26.5%). Interestingly, two-thirds of participants (68.9%) have recommended SM in case of minor illnesses only, 85.3% of respondents have expressed their desire to learn more about appropriate SM, and 76.6% are willing to return their leftover or unused medications to drug take-back centers.

## Introduction

“An attribute which differentiates man from animals is the wish to take medicine,” as stated by William Osler (1). Everyone practices Self-care (SC), and it's not new. The world health organization (WHO) defines self-care as “The ability of individuals, families and communities to promote health, prevent disease, maintain health, and cope with illness and disability with or without the support of a healthcare provider” (2). SC is a key element for better health outcomes and existed well ahead of formal health systems. SC remains a key factor for maintaining health, although healthcare systems do exist (3). Every individual exercise Self-medication (SM) as a part of SC of their health (4). The activity pratctised individually or directed by someone to handle the minor health illnesses is regarded as SM. This definition spells out that all medications cannot be taken on one's own initiative, doctor's prescription is necessary for some medications and medical supervision is essential and clarifies that there is a rational position for SM in developed societies (5). Using leftover medications or medications received from family and friends, including over the counter (OTC) drugs, is considered SM practice (6).

Although SC and SM are essential components of healthcare provision, SM poses benefits and risks. The United States Food and Drug Authority (FDA) defines OTC medicines as “drugs that are safe and effective for use by the general public without seeking treatment by a health professional” (5). Worldwide, it is an acceptable practice to use OTC medications without a prescription (4). As per the WHO's statement, rational SM has numerous benefits in managing several minor health conditions which doesn't require prior medical consultation. Furthermore, SM is an inexpensive replacement that provide several benefits to the patients including increased accesses to treatment, reduce treatment cost and visit to doctors hence decreases the load on health care facilities (5).

Despite all this, irrational and irresponsible SM comes with countless adverse health consequences including low drug efficacy, resistance and tolerance to certain medicines, extreme side effects, drug intolerance, mask the actual diagnosis, numerous drug and food interactions, dependence, under or overdose of drugs, toxicity of drugs and withdrawal symptoms (7). Moreover, inappropriate practice of SM possess numerous potential risks for the individual consumer including incorrect self-diagnosis, failure to seek appropriate medical advice promptly, incorrect choice of therapy, inability to identify certain pharmacological risks, some rare but sever adverse effects, failure to detect contraindications, warnings, precautions and interactions, failure to identify that same medication is consumed under different brand name, failure to report present SM to the prescribing physician, wrong rout or frequency of administration, unnecessary prolonged use, incorrect or prolonged storage of medication than specified by the manufacturer or dispenser, drug induced disease or hospitalization and wasting of public health expenditure. Therefore, the SM should be practiced with the aim of maximizing the benefits and minimizing the above-mentioned risk and adverse consequences (5).

Such preventable adverse consequences of irrational SM practices should be highlighted to the community and acquiring an educational approach toward health education to curb it. The inappropriate practice of SM is increasing steadily in developing countries, approximately as developed countries, prevailing it as a public health concern (8). Apart from harming the patient's health, inappropriate SM also increases treatment cost and frequency of hospital admission.

WHO estimates a projected shortfall of 18 million health workers by 2030, mostly in low- and lower-middle-income countries (9). Hence some countries encourage their residents to adopt SC behavior to manage trivial illnesses including SM. The Responsible SM helps reduce the cost of treatment, traveling time, and consultation time (4, 10). While SM practice's commonness varies from one country to another, the familiar factor that influences SM includes age, gender, income, educational level, medical knowledge, and trivial illnesses (11, 12). Numerous studies have reported the following factors as common reasons to indulge in SM practice; minor illness, previous experience with similar health problems, financial limitations, unavailability of healthcare, waiting time in healthcare facilities, and easy access to OTC drugs (13, 14). Nevertheless, the growing trend of advertisements by pharmaceutical companies, media, and the Internet has largely promoted SM behavior (12, 15). In Saudi Arabia, SM is practiced by all ages, including students, teenagers, adults, and parents (14, 16, 17).

Several studies conducted across the world, including Bangladesh (18), India (19), Ethiopia (20) and Egypt (21), have highlighted the factors contributing to the promotion of SM practice and identified the key factors responsible for irrational SM. Various national studies targeting people and patients who visited community pharmacies, primary healthcare centers and hospitals have been conducted to explore the prevalence of SM (22–25). A few studies were conducted to determine SM with antibiotics (26). The previous **two** SM studies conducted in Riyadh city almost a decade ago (24, 25) mainly targeted people who attended primary health care centers and community pharmacies. In both studies, male participation was dominant. We conducted this study to determine the prevalence of SM and its pattern among all residents above 18 years of age residing in Riyadh city. Additionally, efforts were made to gather information about frequency of SM during last **3** months, source of drug information, place of obtaining medications, outcomes of SM and reason influencing such practice along with their opinions, recommendations, and impact of covid-19 on SM practice.

## Materials and Methods

### Study Design and Population

The present community-based cross-sectional study was carried out to explore the prevalence of self-medication, factors, and suggestions to limit irrational SM practice. The study was conducted between September and October 2021 among residents aged 18 and above of Riyadh, Saudi Arabia. The institutional review board of AlMaarefa University (UM) approved the study with the registration number (02-20102021). The study population was divided into 5 geographical areas in Riyadh city: North, South, East, West, and Central. The residents under 18 years of age, residing outside of Riyadh, staying in Saudi Arabia on visit visas and students of UM (in recent past, SM study has been conducted on UM students) were excluded from the participation. The informed consent was displayed at the beginning of the online survey and the study purpose. Participation was voluntary; they had participation option to choose either agree or disagree. Those who selected disagree option were directed to decline the participation section and finish participation.

### Approach to Convey the Study Purpose and Its Criteria to Participants Effectively

We tried to convey our study's purpose, significance, and criteria through a short video clip [https://youtu.be/KC-E8WiGxjY]. The video clip was uploaded at the beginning of the study and the consent form. Considering that in-person interaction and briefing about the work was not possible with all participants, residents may be reluctant to have any face-to-face interaction due to ongoing Covid-19 pandemic. Lastly, it may be convenient for low educated and elderly participants to refer to video rather than reading. Watching the video was not mandatory; participants could skip the video and attempt the survey if they wish to do so. This approach describes the study purpose and encourages the residents to participate.

### Sample Size and Sampling Method

We calculated the sample size by estimating the total population of Riyadh in 2021 to be 7,387,817 as reported by World Population Review [https://worldpopulationreview.com/world-cities/riyadh-population]. Sample size was calculated using Raosoft sample size calculator by presuming a 95% confidence level, 5% margin of error, and precision level of 5%, yielding a sample size of 385. We adopted convenience and snowball sampling techniques to collect the data.

### Questionnaire Construction

The questionnaire was developed based on a review of relevant literature. We referred three local (Riyadh, Hail and Abha) and four international (India, Bangladesh, Egypt and Ethiopia) studies addressing our topic of interest among community and educational institutes to draft the study tool (14, 17–22). The study tool was subjected to extensive revision to fit the characteristics of the study population. A self-administered questionnaire was categorized into sections. The first section addressed sociodemographic information about participants such as gender, age, education, occupation, marital status, number of children at home, distance to nearest hospital and pharmacy, health insurance, family member working in the health sector, etc. The second segment measured the SM pattern including SM practice during the last 3 months, frequency and outcome of SM, common medical illnesses for which SM was practiced, type of drug used, source of drug information, place of obtaining the drug. The third sections measured reasons influencing SM practice, the negative impact of inappropriate SM and adverse outcome of SM and steps taken to overcome such events. The fourth part was developed to record the participants' opinions toward SM, including their recommendation, willingness to return unused medications, spreading awareness, and the impact of Covid-19 on SM practice.

### Validity, Reliability of Study Tool and Data Collection

The experts from the college of pharmacy, community Pharmacist and Epidemiologist completed the review and evaluation of the questionnaire draft to ensure content validity. The questionnaire was translated into Arabic language by independent professional translator and then back translated to English version through another professional translator to ensure the similarity. The final questionnaire was displayed online bilingually. The face validity was tested by conducting a pilot study on 25 residents (5 participants were randomly selected from each region of Riyadh city), aged between 20 and 50 years. The comments from pilot respondents were, to reduce the number of questions, to simplify the English terminologies for drug class such as mentioning Fever lowering drugs instead of antipyretics and number of children part in the socio-demographic section was changed to a number of children under 18 years of age staying with you, because some respondents mentioned they have children but not staying with them in the city, so that may not have any influence on their SM practice. The data of pilot participants was not included in the final analysis. Moreover, questionnaire reliability was measured by calculating the Cronbach's alpha factor for the questions addressing prevalence, frequency, health outcome, adverse consequences and their management and viewpoint toward self-medication (0.82). The social media platforms and personal contacts were used to distribute the online questionnaire.

### Statistical Analysis

The collected responses were evaluated for their completeness and consistency. Disagreed and incomplete responses were excluded from the analysis. The SPSS (version 27.0, IBM, New York, NY, USA) was used to analyze the data. Cross-tabulation was performed by applying the chi-square test. The odds ratio was calculated to estimate the risk of SM practice; Pearson's correlation was done to establish the link between opinions with SM practice during the last 3 months. Multivariant logistic regression was performed to find the relationship between independent predictors and SM practice. A *p*-value of <0.05 was considered significant.

## Results

### Sociodemographic Details

Thankfully, 91 participants viewed the video clip uploaded at the beginning of questionnaire, indicating it's usefulness. Overall, 625 residents attempted the survey link, out of which 14 disagreed to participate. Hence a total of 611 responses were subjected for data analysis. Major participants were from North of Riyadh (200), followed by Central (110), East (108), West (101) and South parts of the city. Highest participants were females (71.4%); unmarried (52.4%); Saudi citizens (88.4%) and staying with family (89.7%). Regarding Covid-19 vaccination, 545 (89.2%) took two vaccine doses (Table 1).

**Table 1:** Socio-Demographic characteristics of the study participants.

**Demographic variables**		**Number (%)**
Gender	Male	175 (28.6)
	Female	436 (71.4)
Age	18–24 years	231 (37.8)
	25–44 years	221 (36.2)
	45–60 years	135 (22.1)
	61 years and above	24 (3.9)
BMI	Underweight	38 (6.2)
	Healthy	241 (39.4)
	Overweight	191 (31.3)
	Obese	141 (23.1)
Marital Status	Unmarried	320 (52.4)
	Married	273 (44.7)
	Divorced/separated	18 (2.9)
Number of children at home	No Children	217 (35.5)
	1 child	104 (17.0)
	2 Children	98 (16.0)
	3 Children	76 (12.4)
	4 Children	63 (10.3)
	5 Children	26 (4.3)
	>5 children	27 (4.4)
Nationality	Saudi	540 (88.4)
	Non-Saudi	71 (11.6)
Residential status	Living with Family	548 (89.7)
	Living Alone	63 (10.3)
Education level	Pre-high school	16 (2.6)
	High school	169 (27.7)
	College Graduate	360 (58.9)
	Postgraduate	66 (10.8)
Health Insurance	Yes	281 (46)
	No	330 (54)
Physical activity	Yes	290 (47.5)
	No	321 (52.5)
Distance to nearest hospital	<1 km	93 (15.2)
	1–5 km	182 (29.8)
	5–10 km	160 (26.2)
	more than 10 km	176 (28.8)
Distance to nearest Pharmacy	<1 km	350 (57.3)
	1–5 km	213 (34.9)
	5–10 km	34 (5.6)
	more than 10 km	14 (2.3)
Occupation	Student	200 (32.7)
	Home maker	79 (12.9)
	Retired	66 (10.8)
	Health care	66 (10.8)
	Education sector	81 (13.2)
	Sale and business	22 (3.6)
	Construction	1 (0.2)
	IT and telecom	8 (1.3)
	Bank and account	5 (0.8)
	Human Resources	10 (1.6)
	Others	73 (11.9)
Residing in Saudi Arabia since	By birth	542 (88.7)
	5 years	29 (4.7)
	10 years	7 (1.1)
	more than 10 years	33 (5.4)
Family member in Health sector	Yes	272 (44.5)
	No	339 (55.4)
Chronic disease	No	449 (73.5)
	Yes	159 (26.0)
Covid-19 vaccine status	Two doses of vaccine	545 (89.2)
	One dose of Vaccine	46 (7.5)
	Didn't Receive Vaccine	20 (3.3)
Covid-19 Infection status	Active	7 (1.1)
	Recovered	131 (21.4)
	Didn't get covid-19 infection so far	473 (77.4)

### Prevalence of Self-Medication During Last 3 Months

The prevalence of SM is increasing globally, and its rational practice warrant the benefits. Overall, 52.9% have practiced SM during the last 3 months. About one-fourth of participants (24.9%) have practiced SM just one time during the last 3 months. More than two-thirds of participants (72.5%) stated that their health condition improved after practicing SM. About eighty-nine (14.6%) residents mentioned that they experienced some drug-related problems after practicing SM. Out of these, fifty-five (9%) stated they stopped taking medications, and 2% switched to another drug to overcome the drug-related problems (Table 2).

**Table 2:** Self-medication pattern among residents of Riyadh, Saudi Arabia.

**Self-medication practice information**	**Sample *(N)***	**Percentage**
Did you practice SM during last 3 months?		
Yes	323	52.9
No	288	47.1
Frequency of practice in last 3 months		
1 time	152	24.9
2 times	73	11.9
3 times	48	7.9
4 times	25	4.1
5 times	14	2.3
More than 5 times	11	1.8
After practicing SM, my health condition		
Improved	443	72.5
Didn't change	157	25.7
Worsened	11	1.8
Have you experienced any drug related problem after practicing SM?		
Yes	89	14.6
No	522	85.4
What step did you take after experiencing drug related problem due to SM?		
Stopped taking medication	55	9
Switched to another medication	13	2.1
Went to hospital	11	1.8
Reported to person who gave medication	10	1.6
Not experienced any problem due to SM	522	85.4
Place of obtaining the medication for self-use		
Pharmacy store	545	89.2
Family/friends	125	20.5
Unused/left over medicine at home	97	15.9
Internet/online	61	10
Supermarket	36	5.9
Source of drug information		
Previous prescription	283	46.3
Pharmacist	244	39.9
Internet	174	28.5
Friends/relatives	162	26.5
Parents	150	24.5
Advertisements	22	3.6
Newspaper and magazine	14	2.3
Negative impact of inappropriate SM		
Drug side effect	428	70
Drug and food interaction	209	34.2
Poor treatment outcome	199	32.6
Symptoms return	162	26.5
No negative impact	80	13.1

### Source of Information and Place of Obtaining the Drugs for SM

Sources utilized to obtain drug information and to collect the medications influence the appropriateness of SM practice. Overall, half of the participants mentioned previous prescriptions (46.3%) and pharmacists (39.9%) as a main source to obtain drug information. Newspapers, magazines (2.3%) and advertisements (3.6%) were the least referred sources to obtain drug information. Almost 90% of respondents mentioned a Pharmacy store as a prime place to obtain drugs for SM, followed by family & friends (20.5%) and unused/leftover medications (15.9%). Interestingly, respondents reported drug side effects (70%), Interaction (34.2%), poor treatment outcome (32.6%) and return of symptoms (26.5%) as significant adverse consequences of practicing inappropriate SM (Table 2).

### Illnesses and Class of Drugs Used to Practice SM

**Figure 1:**
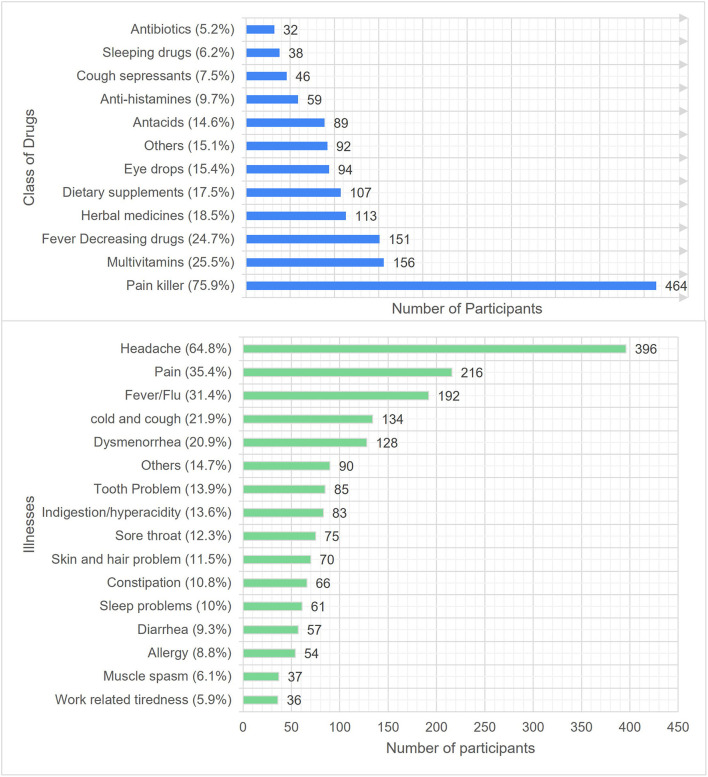
Illnesses and classes of drugs used to practice SM during last 3 months.

Use of OTC medications to manage the trivial illnesses is an indication of rational SM practice. Furthermore, good level of awareness regarding consequences of inappropriate SM encourages the individual consumer to practice SM consciously and seek essential medical advice. The Figure 1 depicts type of illnesses and classes of drugs used to practice SM. Most respondents have reported using pain killers (75.9%), multivitamins (25.5%), antipyretic (24.7%), herbal medicines (18.5%) and dietary supplements (17.5%) to self-treat the illnesses, including headache (64.8%), pain (35.4%), fever/flu (31.4%), cold & cough (21.9%) and dysmenorrhea (20.9%) during last 3 months. On the other hand, the use of antibiotics (5.2%) and sleeping aids (6.2%) were the least commons. The use of other drugs (15.1%) includes anti-emetics, anti-inflammatory, laxatives and topical medications. Similarly, other illnesses (14.7%) include vomiting, mouth ulcer, stress, eye infections and bodybuilding.

### Reasons Impacting SM Practice

**Figure 2:**
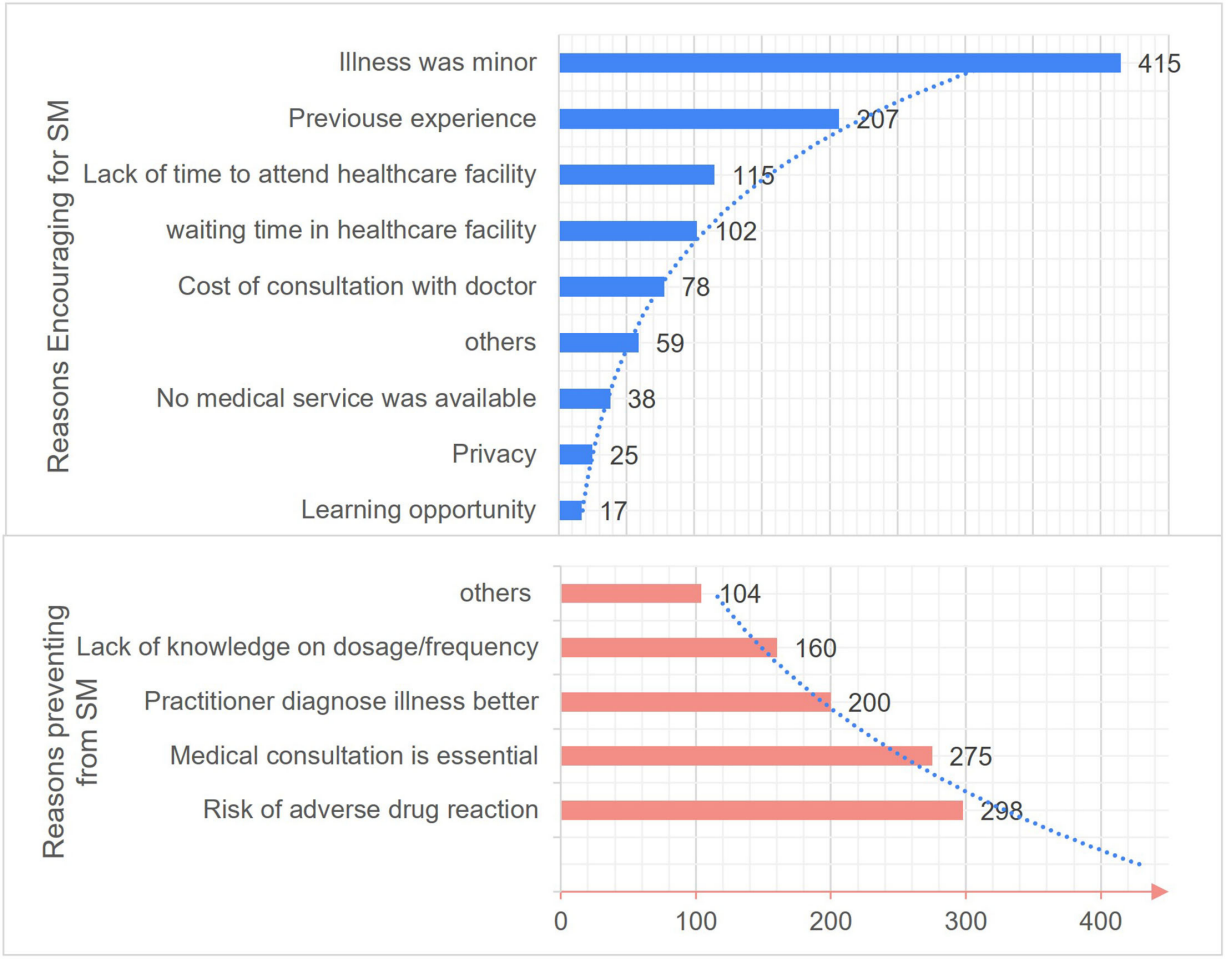
Reasons impacting self-medication practice among Riyadh residents.

The reasons modulating SM practice are depicted in Figure 2. The most common reasons reported by residents as encouraging factors to practice SM includes minor illness (67.9%), earlier experience (33.9%) and shortness of time to attend healthcare facility (18.8%). Excitingly, only a few (2.8%) respondents mentioned that they practiced SM as a learning opportunity. Contrastingly, the typical reasons preventing from practicing SM were risk of adverse reactions (48.8%), the importance of medical consultation (45%), believing in medical practitioner's ability of illness diagnoses (32.7%) and lack of knowledge on dosage and frequency (26.2%).

### Correlation Between SM Practice With Opinions

Pearson correlation analysis revealed the significant positive correlation between SM during last 3 months and opinions 1, 2 and 5. It indicates that those with SM-3 recommend others to practice SM, would like to learn more about SM and assume that covid-19 has increased the practice of SM. Interestingly, all the opinions were positively correlated with each other (Table 3).

**Table 3:** Pearson correlation between opinions with the SM practice.

**Variable**	**SM-3**		**Opinion 1**		**Opinion 2**		**Opinion 3**		**Opinion 4**		**Opinion 5**	
	***r***	***P*** **value**	***r***	***P*** **value**	***r***	***P*** **value**	***r***	***P*** **value**	***r***	***P*** **value**	***r***	***P*** **value**
SM-3	–	—	0.269^**^	0.000	0.181^**^	0.000	0.012	0.760	0.057	0.160	0.187^**^	0.000
Opinion 1			—	—	0.273^**^	0.000	0.166^**^	0.004	0.061	0.130	0.118^**^	0.003
Opinion 2					—	—	0.283^**^	0.000	0.268^**^	0.000	0.105^**^	0.009
Opinion 3							—	—	0.301^**^	0.000	0.108^**^	0.008
Opinion 4									—	—	0.187^**^	0.000

*r = Correlation coefficient*.

** *Significant correlation at the 0.01 level (2-tailed)*.

*SM-3 = Self-medication practice during last 3 months*.

*Opinion 1: Do you recommend self-medication to others?*

*Opinion 2: I would like to learn more about proper self-medication practice*.

*Opinion 3: Are you willing to return your unused/leftover medications if drug take-back centers are made available?*

*Opinion 4: Do you believe that spreading awareness and education regarding the implications of self-medication would limit the inappropriate practice of self-medication?*

*Opinion 5: What is your opinion about the impact of a covid-19 pandemic on self-medication practice?*

### Factors Influencing Self-Medication Practice

Binary logistic analysis was performed to identify the factors influencing SM practice. We didn't observe any significant impact of gender, nationality, residence status, children at home, and family members working in the health sector. Conversely, performing routine physical activity and distance to the nearest hospital from residence showed a significant (*p* = 0.013 and *p* = 0.02) impact on SM practice. The residents who were not performing routine physical exercise had 0.6 times high risk of indulging in SM practice than those who performed routine physical activity. Similarly, participants whose residence was more than 10 km away from the healthcare facility have a 1.7 times higher risk of practicing SM than residents staying near the healthcare facility (Table 4).

**Table 4:** Factors influencing self-medication practice.

		**Self-medication practice during last 3 months**		*p* **-Value**	**Odds ratio (Risk estimate)**		
**Category**	**Subgroup**				**95% confidence interval**		
		Yes (%)	No (%)		Value	Lower	Upper
Gender	Male	101 (16.5)	74 (12.1)				
	Female	222 (36.3)	214 (35)	0.128	1.316	0.924	1.874
Hospital distance from residence	<10 km	247 (40.4)	188 (30.8)	0.02^*^	1.729	1.214	2.462
	More than 10 km	76 (12.4)	100 (16.4)				
Routine physical activity	Yes	138 (22.6)	152 (24.9)	0.013^*^	0.667	0.485	0.919
	No	185 (30.3)	136 (22.3)				
Family member in healthcare	Yes	152 (24.9)	120 (19.6)	0.181	1.244	0.903	1.714
	No	171 (28)	168 (27.5)				
Children at home	No	120 (19.6)	97 (15.9)	0.371	1.164	0.835	1.623
	Yes	203 (33.2)	191 (31.3)				
Nationality	Saudi	280 (45.8)	260 (42.6)	0.167	0.701	0.423	1.162
	Non-Saudi	43 (7)	28 (4.6)				
Residential status	Living with family	292 (47.8)	256 (41.9)	0.539	1.177	0.699	1.984
	Living alone	31 (5.1)	32 (5.2)				

The variables showing a significant correlation in binary logistic regression were put through multivariant regression analysis to determine individual predictors of SM practice. After fine-tuning potential confounding factors, the distance to healthcare facility from residence, and routine physical activity were significant independent predictors of SM (Table 5). The participants who reside at <10 km to healthcare facility have 0.55 times less likelihood of practicing SM than participants who reside more than 10 km from a health care facility. Surprisingly, residents who perform routine physical activity were predicted to practice SM 1.5 times more than those who don't perform any routine physical activity.

**Table 5:** Multivariant logistic regression analysis identifying the variables associated with the self-medication during the last 3 months.

**Independent variables**	**Variable coefficient (B)**	***p*** **-value**	OR (95% CI) Adjusted^*^
*SM practice during last 3 months (YES)*			
Distance to healthcare facility			
<10 km from residence	−0.586	0.001	0.557 (0.389–0.797)^*^
>10 km from residence	–	–	1.00
Routine physical activity			
Yes	0.424	0.011	1.528 (1.103–2.116)^*^
No	–	–	1.00

### Opinions and Recommendations Toward Self-Medication

We collected a few opinions from our study participants at the end of this questionnaire. Interestingly, two-thirds of participants (68.9%) have recommended SM practice to others in case of minor illnesses only. Likewise, most respondents (85.3%) have expressed their desire to learn more about appropriate SM practices. Similarly, most respondents (76.6%) are willing to return their leftover or unused medications to drug take-back centers. Most residents (89%), believe that spreading awareness about the implications of SM would rationalize and improve the SM practice. Expectedly, about 70% of participants assume that the emergence of covid-19 infection has led to increased SM practice (Table 6). Our study noticed a significant difference in opinions 1 and 3 among different age groups.

**Table 6:** Opinions and recommendations toward self-medication.

**Statement**	**Opinions**	**Age in years**				**Total (%)**	***P*** **value**
		**18–24 (%)**	**25–44 (%)**	**45–60 (%)**	**>61 (%)**		
Do you recommend self-medication to others?	Always	21 (3.4)	17 (2.8)	6 (1)	2 (0.3)	46 (7.5)	0.020^*^
	Only for minor illnesses	172 (28)	138 (22.5)	97 (15.9)	14 (2.3)	421 (68.9)	
	No	38 (6.2)	66 (10.8)	32 (5.2)	8 (1.3)	144 (23.6)	
I would like to learn more about proper self-medication practice	Yes	197 (32)	185 (30.2)	118 (19.3)	21 (3.4)	521 (85.3)	0.798
	No	34 (5.6)	36 (5.9)	17 (2.8)	3 (0.5)	90 (14.7)	
Are you willing to return your unused/leftover medications if drug take-back centers are made available?	Yes	158 (25.9)	171 (28)	116 (19)	23 (3.8)	468 (76.6)	0.001^*^
	No	73 (11.9)	50 (8.1)	19 (3.1)	01 (0.2)	143 (23.4)	
Do you believe that spreading awareness and education regarding the implications of self-medication would limit the inappropriate practice of self-medication?	Yes	201 (32.9)	195 (31.9)	127 (20.8)	21 (3.4)	544 (89)	0.197
	No	30 (4.9)	26 (4.2)	8 (1.3)	3 (0.5)	67 (11)	
What is your opinion about impact of covid-19 pandemic on self-medication practice?	Increase in SM practice	165 (27)	165 (27)	87 (14.2)	15 (2.5)	432 (70.7)	0.133
	Decrease in SM practice	16 (2.6)	15 (2.5)	18 (2.9)	01 (0.2)	50 (8.2)	
	No impact	50 (8.2)	41 (6.7)	30 (4.9)	8 (1.3)	129 (21.1)	

## Discussion

Although SM is a part of self-care and supported by WHO to manage numerous minor illnesses that don't require medical consultation, its effectiveness is primarily determined by appropriateness and rational use. Hence inappropriate and irrational SM leads to dosage and treatment errors, adverse drug events, and risk of addiction or abuse. Although SM poses numerous advantages, yet it's largely determined by who uses it and how it is practiced.

The earlier two studies conducted in Riyadh to address the SM issue were “Self-medication in central Saudi Arabia: community pharmacy consumers perspective” in 2011 (25) and “Self-medication practice among patients in a public health care system” in 2009 (24). The 2011 study targeted people visiting community pharmacies in Riyadh, whereas a 2009 study was conducted on patients attending primary healthcare centers. However, our study was aimed at all the residents of Riyadh, from age 18 years and above.

Additionally, our study gathered information regarding Covid-19 infection and vaccine's impact on SM, opinion, and recommendations of residents about SM and adverse outcome of SM and their actions to resolve those outcomes. Our study shows that respondents who had practiced SM during the last 3 months were 323 (52.9%), which was lower compared (81.3%) to the regional study of SM for oral health among adults of Riyadh (63.25%) (27), residents of Medina city (28) and international studies from Savar residents (60.2%), in Bangladesh (18), Addis Ababa community (75.5%) Ethiopia (20), people attending oral health program in Tamil Nadu (69.32%) India (19), patients attending El-Mahsama family practice clinic, Ismailia (96%) Egypt (21), a community of Karachi (84.8%) Pakistan (29) and higher (45.4%) compared to the residents of Wuhan, China (30).

Correlating our outcomes with earlier studies (18, 23, 25, 30), where SM was significantly influenced by age, gender, education, occupation and marital status, our study found a significant impact of physical activity and distance to a healthcare facility on the practice of SM. Most respondents (72.5%) stated that their health condition improved after practicing SM, a similar outcome was noted among Addis Ababa Community Ethiopia (20). About half of respondents (55.5%) consider SM as a safe exercise, which is higher compared to the findings from residents of Hail (22), Saudi Arabia (33.5%).

The most common illnesses for which SM was practiced includes headache (64.8%), pain (35.4%), fever/flu (31.4%) and cold and cough (21.9%). Similar findings have been noted in earlier studies (19–23, 28–30). The typical classes of drugs used to practice SM were pain killers (75.9%), multivitamins (25.5%) and antipyretics (24.7%), which are in parallel with the findings of previous studies (19–21, 27, 28) and community pharmacy consumers of Al-Qassim province (31).

Interestingly, antibiotics were the least (5.2%) commonly used medication to practice SM, which are contrary to the finding from Egypt (21) (23.5%), antibiotic use in Saudi Arabia (26) (43.4%), Medina (28) (15.3%), SM for oral health among adults (27) of Riyadh (17.79%) and Qassim (31) (13.8%). Whereas study conducted on a community of Karachi, Pakistan (29) showed much lower (1.2%) use of antibiotics for self-care compared with our findings. Surprisingly, about 18.5% of our participants used herbal products for SM, which are like the findings of Addis Ababa (20) (16%). Likewise, 17.55 of participants used dietary supplements for self-care. The use of such conventional products would be related to the ongoing Covid-19 phase, reluctance to visit healthcare facilities and recommendations from family and friends.

The reinforcing reasons for SM practice mentioned in our study were trivial illness, previous experience, lack of time and waiting time in healthcare facilities like earlier studies conducted in Riyadh. Similar findings were observed in earlier studies (18–20, 28, 30, 31). Whereas studies conducted in Karachi, Pakistan (29) and Ismailia, Egypt (21) indicated convenience, cost-saving and previous experience as major reasons to opt for SM practice. Interestingly, few (2.8%) participants practiced SM as a learning opportunity, which indicates their responsible behavior toward medications and SM. About 47% of residents haven't practiced any SM during the last 3 months. The most common reasons which discouraged them were the risk of adverse drug reaction (48.77%), feeling that medication consultation is necessary (45%), and expertise of medical practitioners in disease diagnosis (32.7%). Parallel to our finding, the study conducted among a community of Addis, Ababa, Ethiopia (20) also found fear of wrong diagnosis, wrong drug and side effects as typical reasons which discouraged SM practice.

Easy availability of medications may encourage SM. The frequent place for obtaining medications for self-care was pharmacy store (89.2%), followed by borrowing medicines from family & friends (20.5%) and use of leftover medications (15.9%). Similar observations were noted among the studies conducted in Bangladesh (18), Addis Ababa (20), rural population in South-western Saudi Arabia (23) and residents of Karachi city, Pakistan (29). Although some OTC medications are available at the supermarket in Saudi Arabia, only a few residents (5.9%) preferred to buy them from Non-pharmacy stores. This behavior indicates the importance of pharmacist and their advice on SM. We noticed that residents of Riyadh city relied on previous prescriptions (46.3%), pharmacists (39.9%), the Internet (28.5%) and family/friends (26.5%) to get the information about medications which they prefer to self-medicate. These findings are in line with earlier study outcomes (20, 21, 25, 28, 30).

Irrational SM not only leads to an incorrect diagnosis but is also a risk factor for exacerbation of disease and severe health consequences. Residents of Riyadh city have depicted consciousness regarding the negative impact of irresponsible SM practice. The most common impact of inappropriate SM stated is a drug side effect (70%) followed by drug interactions (34.2%), poor health outcome (32.6%) and return of symptoms (26.5%). The participants of this study have shared their opinions and recommendations related to SM. About two-thirds (68.9%) of residents have recommended SM practice only for minor illnesses. Likewise, 85.3% have expressed their wish to return unused/leftover medications to drug take bac centers. The majority (85.3%) are willing to learn more about proper SM, whereas 89% recommend spreading awareness about rational SM practice. Eventually, respondents have also mentioned their view on the impact of ongoing Covid-19 on SM practice. About 70.7% of study participants believe that SM practice has increased during this Covid-19 phase.

### Strengths and Limitations

Although we needed a sample of 385, we could gather 611 responses from all the regions of Riyadh. We restricted the recall questions (“Did you practice SM during last 3 months?” and “Frequency of practice in last 3 months”) to the last 3 months in the SM practice section to avoid recall bias and get a more realistic return. Because in-personal interaction and briefing about the work are not possible with all participants, residents may be reluctant to have any face-to-face interaction due to the ongoing Covid-19 pandemic. Lastly, it may be convenient for low educated and elderly participants to refer to video rather than reading. We tried to convey our study's purpose, significance, and criteria through a short video clip. The video clip was uploaded at the beginning of the study along with the consent form. We approached the residents through convenient and snowball sampling techniques.

## Conclusion

SM has been practiced by about half of the respondents in the recent 3 months. Interestingly, the majority have utilized over-the-counter medications to treat minor and self-diagnosable ailments. Most of them got their medications from a pharmacy and learned about the drugs from the pharmacist. Furthermore, residents have demonstrated a high level of awareness of the detrimental consequences of irrational SM. Only common mild ailments should be treated with SM, according to respondents. They have also stated a desire to return their unneeded and leftover drugs to drug take-back centers, thereby curbing inappropriate SM and decreasing drug storage responsibilities at homes as well as wasteful medication borrowing among friends and relatives. For promoting rational self-medication practices, patient health awareness programs, community pharmacist assistance, continuing medical education programs for health care providers, and planned interventions in the media, such as newspapers, magazines, and television are required.

## Data Availability Statement

The original contributions presented in the study are included in the article/supplementary material, further inquiries can be directed to the corresponding author.

## Ethics Statement

The studies involving human participants were reviewed and approved by the Institutional Review Board of AlMaarefa University (UM) approved the study with the registration number (02-20102021). The patients/participants provided their written informed consent to participate in this study.

## Author Contributions

BM, SAA, and IS devised the project and conceptualized the ideas. JA, MSA, and AA-Q were involved in the literature review. SMBA, NO, and SOA participated in the study design and evaluation of the questionnaire. BM, SMBA, and SAA assisted in data collection and data entry. BM and MYA performed data analysis and drafting of the manuscript. NO, JA, and AA-Q contributed substantially to manuscript revision. All authors have read and agreed to the published version of the manuscript. All authors contributed to the article and approved the submitted version.

## Funding

The authors are thankful to the Deanship of Scientific Research, Najran University, Najran, Saudi Arabia, for supporting this research through grant research code NU/RC/MRC/11/3.

## Conflict of Interest

The authors declare that the research was conducted in the absence of any commercial or financial relationships that could be construed as a potential conflict of interest.

## Publisher's Note

All claims expressed in this article are solely those of the authors and do not necessarily represent those of their affiliated organizations, or those of the publisher, the editors and the reviewers. Any product that may be evaluated in this article, or claim that may be made by its manufacturer, is not guaranteed or endorsed by the publisher.
